# Global Adipose Tissue Remodeling During the First Month of Postnatal Life in Mice

**DOI:** 10.3389/fendo.2022.849877

**Published:** 2022-02-17

**Authors:** Johanna Bruder, Tobias Fromme

**Affiliations:** ^1^ Else Kröner-Fresenius Center for Nutritional Medicine (EKFZ), Technical University of Munich, Freising, Germany; ^2^ Chair of Molecular Nutritional Medicine, TUM School of Life Sciences, Technical University of Munich, Freising, Germany

**Keywords:** white adipose tissue (WAT), white adipose tissue (WAT) browning, brown adipose tissue (BAT), beige adipocyte, postnatal, organ development

## Abstract

During the first month of postnatal life, adipose tissue depots of mice go through a drastic, but transient, remodeling process. Between postnatal days 10 and 20, several white fat depots display a strong and sudden surge in beige adipocyte emergence that reverts until day 30. At the same time, brown fat depots appear to undergo an opposite phenomenon. We comprehensively describe these events, their depot specificity and known environmental and genetic interactions, such as maternal diet, housing temperature and mouse strain. We further discuss potential mechanisms and plausible purposes, including the tempting hypothesis that postnatal transient remodeling creates a lasting adaptive capacity still detectable in adult animals. Finally, we propose postnatal adipose tissue remodeling as a model process to investigate mechanisms of beige adipocyte recruitment advantageous to cold exposure or adrenergic stimulation in its entirely endogenous sequence of events without external manipulation.

## Introduction

Mammalian adipose tissue is a plastic organ with the capacity to dramatically alter size and composition. White adipose tissue (WAT) represents the classical, fat-storing adipose tissue composed of large cells with a single lipid droplet. It also acts as an endocrine organ secreting hormones, such as adiponectin and leptin. Brown adipose tissue (BAT), on the other hand, provides a means of non-shivering thermogenesis in many mammals including adult humans (1–3). It consists of multilocular cells equipped with many mitochondria featuring uncoupling protein 1 (UCP1), the functional core of heat generation. By uncoupling respiration from ATP synthesis, energy of proton motive force is dissipated as heat [reviewed in (4)]. Beyond BAT, UCP1 expressing multilocular cells with high thermogenic capacity are also found interspersed within WAT depots, a cell population called beige or brite adipocytes (5–7).

Mechanisms governing the considerable variability and plasticity of beige cell number are subject to intense research for their potential targeting in humans. Beige and brown adipocyte overall thermogenic capacity is a bottleneck in the efficacy of brown fat targeting drugs in development for the treatment of metabolic disease (8–10).

Well investigated models include cold exposure and application of β-adrenergic agonists in rodents (6, 11). Less studied is a phenomenon that occurs even in the absence of external intervention, i.e. the global remodeling of adipose tissues early in mouse postnatal life.

## Transient, Postnatal Adipose Tissue Remodeling

During the first three weeks of postnatal life, murine adipose tissues undergo a drastic, if transient, remodeling. This phenomenon was first observed in retroperitoneal WAT (rWAT) of male A/J and C57BL6/J mice (12). Here, UCP1 transcript and protein expression sharply increases between postnatal day 10 and 20, only to decrease again towards day 30. A similar transient browning of WAT was again reported for rWAT and inguinal WAT (iWAT) of 1296sv/ev and C57BL6/N mice (13). Thus, a surge in WAT browning takes place around day 20 in multiple fat depots and all studied mouse strains, while in BAT and epididymal WAT (eWAT), UCP1 expression remained unchanged during this period. The extent of this transient remodeling is different by mouse strain and ambient temperature, as comprehensively reviewed below. Our understanding of its exact temporal sequence is limited by the choice of time points studied with low resolution in the past: peak browning has been varyingly detected at day 20 (12–15), several days earlier (16) or later (17, 18).

Interestingly, inguinal and retroperitoneal – but not BAT - depots display arrested growth during browning between postnatal day 10 and 20, indicating a profound, depot-level remodeling event (13). Indeed, visualized by phase contrast computed tomography, virtually all mouse fat depots undergo a significant transient period of remodeling around day 20 (14). The evaluated electron density is a combined (alas inseparable) measure of fat content and mitochondrial density and clearly portrays the strong browning surge in rWAT, iWAT and other white depots. Surprisingly, a concomitant, but reverse, remodeling appears to occur in BAT depots. Since this ‘whitening’ surge in BAT does not include altered UCP1 expression (12), it is likely explained by altered fat, not mitochondrial, content. To date it remains unclear whether concurrent ‘whitening of BAT’ and ‘browning of WAT’ are separate events or aspects of the same epi-phenomenon.

## Adipose Tissue Development and Plasticity

White and brown fat differ in extent and direction of postnatal remodeling as well as in their preceding development. BAT fully develops before birth, as determined by mass, UCP1 transcript and protein expression (12, 19). Immediately at birth, pups have to be able to defend body temperature. After birth, brown adipose tissue still grows, mainly due to proliferation until postnatal day 14 and thereafter by storing more lipid (20), a transition suggestively synchronous to the observed whitening (14).

Compared to BAT, development of WAT is less advanced at birth. The iWAT and rWAT depots are still very small, although development has started in the embryo already (21, 22) and existing adipocytes are essentially functional (22, 23). In the first days, these depots expand quickly (23–25). More delayed, functional gonadal WAT (gWAT) is absent at birth (21–23). The first fully differentiated cells do not appear until day 7 (23). The depot specificity of this developmental timeline matches the pattern of depot specific postnatal remodeling with transient browning in fully differentiated WAT depots, but not in still immature eWAT. Intriguingly, and plausibly a repercussion, adult mouse eWAT is considered the whitest depot of all, containing the least fraction of beige adipocytes.

In the adult mouse, adipose tissue remains an organ with extraordinary plasticity that constantly adapts to environmental challenges. Its most obvious flexibility is the huge expandability by combined hyperplasia and hypertrophy, i.e. by adding more adipocytes and by increasing their triglyceride stores (26). Fat mass increase in response to positive energy balance is predominantly attributable to the largest subcutaneous depot, iWAT. The largest visceral adipose tissue depot, gWAT, also has the capability to store an enormous amount of lipids, but in contrast to iWAT it decreases expansion speed after several weeks of high fat diet feeding (27, 28). In comparison to postnatal remodeling, however, the pattern of expansion potential (high in both gWAT and iWAT, low in rWAT) does not seem to match the proneness to transient browning (high in iWAT and rWAT, low in gWAT).

Another plastic characteristic of WAT in adult mice is the flexible number of interspersed beige adipocytes. The interconversion from white to beige adipocytes is a reversible, adaptive process (29), conferring varying degrees of non-shivering thermogenic capacity (30, 31). Accordingly, beige adipocyte recruitment is intensely studied as putative pharmacological target process in the field of metabolic disease [reviewed in (32)]. In iWAT, cold stimulation leads to a strong increase in the number of beige adipocytes (11). On the contrary, thermoneutrality decreases thermogenic beige cell number in mice (33). Being fully reversible, the process can repeatedly be re-activated anytime by another bout of cold stimulation (34), although to a lesser extent with increasing age (31). Intriguingly, adaptive browning in response to cold in the adult mouse displays a similar pattern as postnatal, transient browning, both on the level of depot specificity and in the proneness of different mouse strains. Either both the postnatal, transient process and its adult, adaptive counterpart are subject to the same underlying preconditions or the former establishes the capacity of the latter. Indeed, ablation of postnatally recruited beige cells impairs cold-induced beige adipocyte formation in the adult animal (18).

## Potential Mechanisms of Adipose Tissue Remodeling

Postnatal remodeling of mouse fat depots is characterized by parallel changes in the abundance of UCP1 transcript, mitochondrial density, fat content, and histological appearance (12–14). Categorically, these changes can be driven by differentiation of new cells with different characteristics or, alternatively or additionally, by the transdifferentiation of existing mature adipocytes. These same two options apply for the second, reverting phase of remodeling and not necessarily to the same extent.

Both mechanisms, stem cell differentiation and transdifferentiation, in principle exist and have been described to contribute to adipose tissue plasticity. The existence of the former is non-controversial as every mature adipocyte necessarily descends from a precursor, i.e. a committed preadipocyte and that in turn from a pluripotent, mesenchymal stem cell (35–37). During maintenance, cellular turnover in adipose tissues is regarded to be low, ~10%/year (38). At the same time, the vast expandability of adipose tissue mass in response to prolonged, positive energy balance showcases the massive capacity to generate new adipocytes when hypertrophy of existing ones is exceeded [reviewed in (39)]. In addition, the quick expansion of certain virtually absent adipose tissue depots directly after birth provides ample support for the possibility of rapid hyperplasia being behind the observed remodeling.

The second option, transdifferentiation of preexisting mature adipocytes, has long been suspected and recently proved to constitute a relevant *in vivo* mechanism of white/beige fat cell conversions (29, 40). During this process, formerly white adipocytes acquire the characteristics of beige adipocytes and vice versa. As of today, it is unknown whether all or most white adipocytes inherently possess this ability or only a subset of ‘camouflaged’, white-appearing beige precursors. In any case, the sudden appearance and disappearance of white versus beige adipocytes during postnatal adipose tissue remodeling would be well in line with a transdifferentiation process and importantly, would as such not require massive proliferation and later apoptosis (41). These two latter processes thereby represent indicators to differentiate between the underlying processes at work, but have not been studied exhaustively in this context. At least as far as proliferation is concerned, virtually all adipocytes present at postnatal day 28 (late in the remodeling phase) seem to have already been present at day 10 (early in the remodeling phase), in murine subcutaneous fat that is prone to browning (21), arguing against progenitor proliferation and differentiation as a significant source of beige adipocytes.

Further available evidence to distinguish differentiation from transdifferentiation is limited to depot mass and volume changes concomitant to postnatal browning/whitening. While admittedly crude proxies, these clearly correlate with adipose tissue remodeling, i.e. depots arrest growth during browning and do not during whitening, both on the level of individual fat depots (13) and as a general trend across all depots (14). Specifically in white fat and far from a final assessment, these observations are in line with predominant transdifferentiation of existing cells during both the browning of white fat and its reversion, possibly accompanied by a diluting effect of newly differentiating cells during the latter phase.

## Potential Physiological Purposes of Postnatal Adipose Tissue Remodeling

Apart from the exact mechanisms at work bringing about postnatal, transient fat browning or whitening, the overarching question certainly pertains to the ‘why’ of this adipose organ-wide phenomenon. Two alternative, principal scenarios are possible: first, adipose tissues are transiently remodeled to serve an acute functional purpose specific during this short period in postnatal development, or second, postnatal adipose tissue remodeling is a preparative phenomenon creating a cellular complexity to be adaptively utilized during adult life. The crucial difference between these is whether those adipocytes that underwent a transient change revert to their original state after fulfilling a transient role or whether they become a new type of cell with perpetually altered adaptive potential.

At three weeks of age, mice are typically weaned and forced to replace a diet of mother’s milk with solid food, a transition with plausible profound effects on metabolism and adipose tissues. Left with the dam, pups still undergo adipose tissue remodeling (13), but must be expected to start nibbling solid food around the same age. How and why this dietary transition would lead to a massive bout of WAT browning seems questionable. More intuitively, the thermoregulatory requirements of small, fur-less mouse pups support a functional role of transient browning in non-shivering thermogenesis. Birth marks a radical transition from the controlled, thermoneutral environment of the womb into a cool world. This plausibly requires a transient extra-capacity of non-shivering thermogenesis that is later alleviated by a rapidly decreasing surface-to-volume ratio and fur growth. Indeed, brown adipose tissue is already fully developed at birth, while non-thermogenic white adipose tissue development occurs mostly postnatally (42, 43).

As plausible as this interpretation sounds, it fails to explain a simultaneous whitening of brown adipose tissues, if these two transitions are in fact causally connected. Both types of adipose tissue undergo postnatal developmental stages possibly accounting for the observed remodeling events: although functional at birth, BAT continues growth by an initial postnatal phase of rapid precursor proliferation and subsequent terminal differentiation including triglyceride loading (20). At the turning point, these events may be misinterpreted as whitening of existing brown adipocytes. In comparison with the adult version of BAT whitening during extended periods of thermoneutrality, postnatal whitening appears to be limited to fat content, not UCP1 abundance, and thus to serve a different purpose (14). Possibly, increased fat content is the consequence of a developmental bout of increased *de novo* lipogenesis, a powerful, cold-stimulated process in mature BAT (44, 45). Similarly, white adipose tissue experiences a distinct postnatal phase of strong sympathetic neurite innervation independent of ambient temperature (16, 17). This may be accompanied by a transient increase in sympathetic tone during the establishment of ligand-receptor connections, in turn underlying an apparent browning.

Importantly, none of these options are mutually exclusive and the causal reason may be distinct from the final one. Browning caused by developmental innervation may at the same time serve the acute purpose of additional thermogenic capacity or create a subset of adipocytes pre-programmed to serve as future beige adipocytes in the adult animal. The existence of the latter, the creation of a separate pool of cells with adaptive potential in the adult animal, is clearly evidenced by reduced, cold-induced browning of adult WAT after ablation of postnatal beige cells (18). Their functional role can be probed by comprehensive mapping of the adult, phenotypic response to a variety of metabolic and environmental challenges after manipulating postnatal browning/whitening surge intensity, as outlined in the following.

## Factors Modulating Postnatal Adipose Tissue Remodeling

Genetic background plays an important role in postnatal adipose tissue remodeling. Several studies established a different susceptibility to transient WAT browning, e.g. lower in C67BL6/J and /N compared to A/J or 129SvEv mice (12, 13, 15). This pattern matches well with the known propensity to adult, adaptive browning (30, 46–48). Phenotypic strain differences offer the chance to identify genetic factors (48), but efforts to identify adult consequences of postnatal adipose tissue remodeling will be superimposed by unrelated differences in genetic outfit. Ideally, postnatal events could be gradually manipulated in genetically identical animals followed by comprehensive mapping of the adult response to metabolic challenges.

The ontogenetic earliness of postnatal adipose tissue remodeling limits the experimental options to manipulate these events to the first days in life or to maternal effects (maternal programming). Luckily, there is evidence for the efficacy of both. The most obvious environmental parameter interacting with the abundance of thermogenic cells is ambient temperature. Indeed, the peak of postnatal browning in iWAT occurs earlier (day 21) and is more pronounced when offspring and dam are housed at 30°C as compared to 22°C (day 28) (17). Furthermore, offspring of dams housed at 17°C during lactation showed higher UCP1 expression in iWAT at postnatal day 21 than of dams at thermoneutrality (15). When exposed to cold as adults, however, temperature early in life did not impact later browning capacity in fat depots in this study. Since ablation of postnatally recruited beige cells did lead to an adult limitation of browning capacity (18), it will be interesting to investigate the extent at which a postnatal manipulation robustly manifests in adult, metabolic consequences in future studies.

An alternative condition to manipulate postnatal adipose tissue remodeling in pups is maternal diet quality and quantity during gestation or lactation. Indeed, undernutrition of pups leads to reduced postnatal browning at postnatal day 21 in iWAT, while overfeeding does not (49). Interestingly, neither affects susceptibility to diet induced obesity later in life or the extent of browning in response to cold exposure. Adult UCP1 expression and thermogenic capacity of BAT, however, is clearly altered in response to manipulated maternal lactation, either by high fat diet feeding or as a function of litter size (50–52). Possibly, a direct effect of these regimes on postnatal BAT remodeling programs this tissue to different states of adult adaptability. And indeed, altered milk quality acutely impacts postnatal adipose tissue remodeling, as demonstrated by supplementation of n-3 polyunsaturated fatty acids to lactating dams (53). This dietary challenge leads to increased BAT UCP1 transcript and protein expression in 21 day old pups. It remains to be tested whether these direct, postnatal effects of lactation are a causal step along the causal route of maternal programming of adult BAT adaptability.

A further approach is based on the development of adipose tissue sympathetic innervation. In adult mice, sympathetic norepinephrine recruits and activates beige/brown adipocytes (54). Sympathetic innervation development and postnatal browning of white adipose tissue have recently been debated to be causally linked (55) or not (17). In any case, from postnatal day 6 onwards, sympathetic innervation and number of beige cells concomitantly increase in iWAT of C57BL6/J mice, until a peak around day 12-16 (16). Importantly, the hormone leptin constitutes the key driver of sympathetic innervation, potentially offering an experimental route to influence postnatal browning in iWAT. Experiments with ob/ob mice, devoid of leptin, revealed less beige adipocytes as well as less dense sympathetic innervation in iWAT, while daily leptin injections between postnatal days 8 and 16 rescued this phenotype (55).

Taken together, ambient temperature and maternal diet are efficient means to manipulate both postnatal adipose tissue remodeling and adult cold response. Initial such experiments report conflicting evidence on a possible causal link between the two.

## Discussion and Outlook

**Figure 1:**
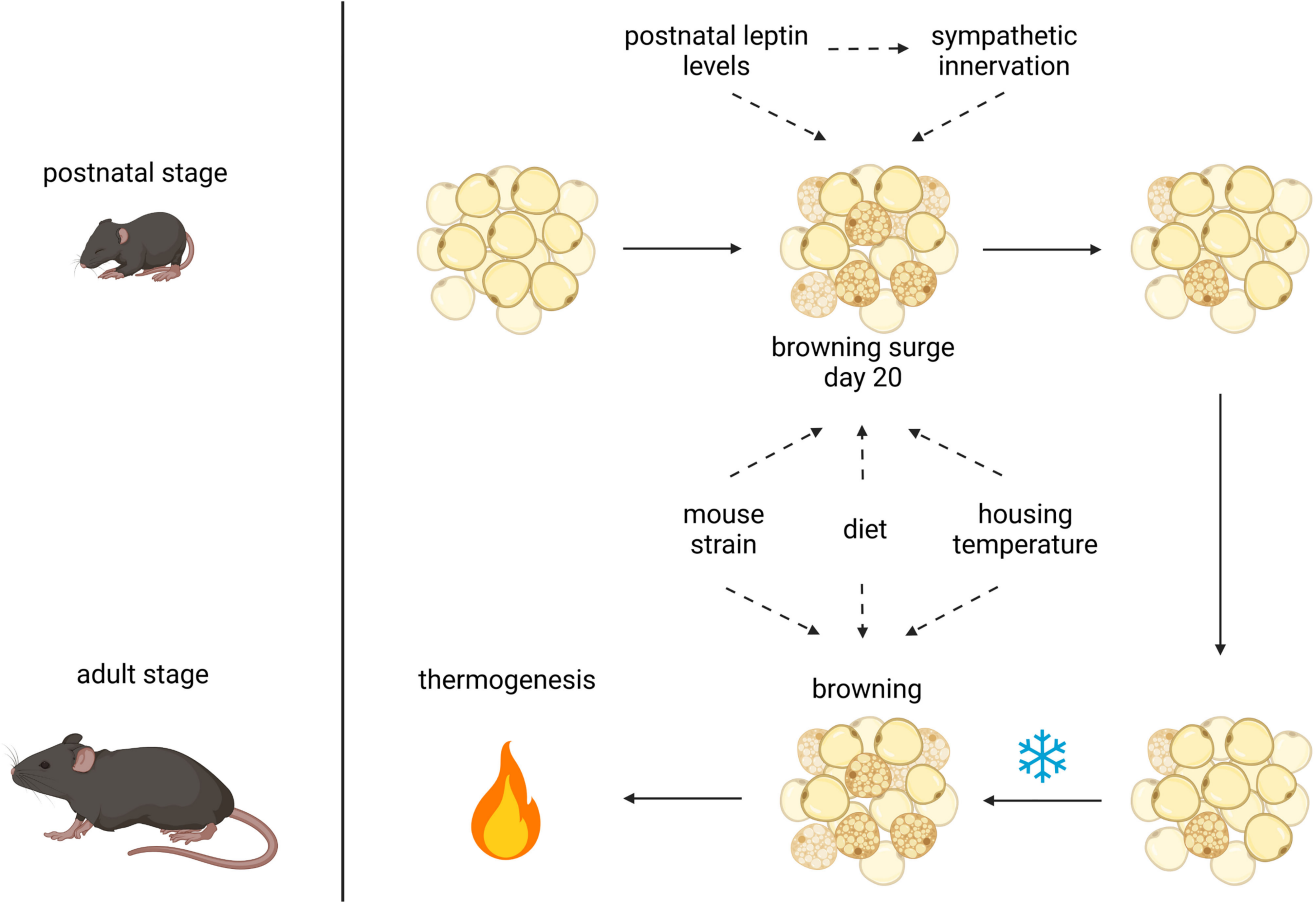
Postnatal and adult adipose tissue remodeling and its possible influencing factors. Browning of white adipose tissue after birth may be affected by leptin levels, sympathetic innervation, mouse strain, maternal diet and housing temperature. The latter 3 factors also have an impact on browning of WAT in the adult animal upon cold stimulation to induce thermogenesis, possibly in concert with postnatally acquired capabilities; created with BioRender.com.

During the past decade, many studies have corroborated postnatal adipose tissue remodeling (12–14, 17, 19, 21, 55) and addressed putative functional aspects [reviewed in (56)] (**Figure 1**). It is now clear that virtually all fat depots, white and brown, simultaneously undergo transient remodeling during the first weeks of postnatal life (14). It is unknown whether ‘whitening in brown’ and ‘browning in white’ fat are independent or linked.

The key question is why these changes occur and whether they serve an acute physiological purpose or are a developmental step in the preparation of later, adult capabilities. Both scenarios can be addressed by similar experimental models, i.e. the targeted manipulation of postnatal events and measurement of acute and delayed impairment of metabolic flexibility. A first step in this direction has already been undertaken by the targeted ablation of postnatally recruited beige cells. This intervention does indeed impair browning capacity later in life (18). Less drastic regimes employing ambient temperature, litter size and maternal diet quality and quantity did, however, not yield unequivocal results so far (15, 17, 50–53, 55). Manipulations of leptin levels constitute additional, experimental opportunities *via* its role during developmental adipose tissue innervation.

Beyond acute thermogenic activation, modulating the capacity and number of brown and beige adipocytes has long been identified a key challenge in their pharmacological exploitation in human metabolic disease. While cold-induced and adrenergic recruitment in rodent models have been intensely studied to identify putative target mechanisms, postnatal adipose tissue remodeling has attracted much less attention - an undeserved neglect in the light of its endogenous occurrence without external intervention, offering an unbiased mechanistic discovery potential.

## Author Contributions

Both authors jointly and equally conceived, wrote and approved the manuscript.

## Funding

JB is supported by the Else Kröner-Fresenius Stiftung (EKFS). TF is supported by the German Research Foundation (DFG; FR 3628/2-1 and TRR333/1 - 450149205).

## Conflict of Interest

The authors declare that the research was conducted in the absence of any commercial or financial relationships that could be construed as a potential conflict of interest.

## Publisher’s Note

All claims expressed in this article are solely those of the authors and do not necessarily represent those of their affiliated organizations, or those of the publisher, the editors and the reviewers. Any product that may be evaluated in this article, or claim that may be made by its manufacturer, is not guaranteed or endorsed by the publisher.

## References

[1] Saito M Okamatsu-Ogura Y Matsushita M Watanabe K Yoneshiro T Nio-Kobayashi J. High Incidence of Metabolically Active Brown Adipose Tissue in Healthy Adult Humans: Effects of Cold Exposure and Adiposity. Diabetes (2009) 58(7):1526–31. doi: 10.2337/db09-0530 PMC2699872 19401428

[2] Van Marken Lichtenbelt WD Vanhommerig JW Smulders NM Drossaerts JMAFL Kemerink GJ Bouvy ND. Cold-Activated Brown Adipose Tissue in Healthy Men. N Engl J Med (2009) 360(15):1500–8. doi: 10.1056/NEJMoa0808718 19357405

[3] Cypess AM Lehman S Williams G Tal I Rodman D Goldfine AB. Identification and Importance of Brown Adipose Tissue in Adult Humans. N Engl J Med (2009) 360(15):1509–17. doi: 10.1056/NEJMoa0810780 PMC2859951 19357406

[4] Klingenspor M Bast A Bolze F Li Y Maurer S Schweizer S. Chapter 4 Brown Adipose Tissue. In: Symonds ME, editor. Adipose Tissue Biology, 2nd. New York: Springer International Publishing (2017). p. 91–147.

[5] Wu J Boström P Sparks LM Ye L Choi JH Giang AH. Beige Adipocytes Are a Distinct Type of Thermogenic Fat Cell in Mouse and Human. Cell (2012) 150(2):366–76. doi: 10.1016/j.cell.2012.05.016 PMC3402601 22796012

[6] Okamatsu-Ogura Y Fukano K Tsubota A Uozumi A Terao A Kimura K. Thermogenic Ability of Uncoupling Protein 1 in Beige Adipocytes in Mice. PloS One (2013) 8(12):1–10. doi: 10.1371/journal.pone.0084229 PMC3875535 24386355

[7] Klingenspor M Herzig S Pfeifer A. Brown Fat Develops a Brite Future. Obes Facts (2012) 5:890–6. doi: 10.1159/000346337 23296106

[8] Gerngroß C Schretter J Klingenspor M Schwaiger M Fromme T. Active Brown Fat During 18F-FDG PET/CT Imaging Defines a Patient Group With Characteristic Traits and an Increased Probability of Brown Fat Redetection. J Nucl Med (2017) 58(7):1104–10. doi: 10.2967/jnumed.116.183988 28104743

[9] Lidell ME Betz MJ Enerbäck S. Brown Adipose Tissue and Its Therapeutic Potential. J Intern Med (2014) 276(4):364–77. doi: 10.1111/joim.12255 24717051

[10] Singh AM Dalton S. What Can “Brown-Ing” Do for You? Trends Endocrinol Metab (2018) 29(5):349–59. doi: 10.1016/j.tem.2018.03.002 PMC5937921 29606342

[11] Lee YH Petkova AP Konkar AA Granneman JG. Cellular Origins of Cold-Induced Brown Adipocytes in Adult Mice. FASEB J (2015) 29(1):286–99. doi: 10.1096/fj.14-263038 PMC4285542 25392270

[12] Xue B Rim JS Hogan JC Coulter AA Koza RA Kozak LP. Genetic Variability Affects the Development of Brown Adipocytes in White Fat But Not in Interscapular Brown Fat. J Lipid Res (2007) 48(1):41–51. doi: 10.1194/jlr.M600287-JLR200 17041251

[13] Lasar D Julius A Fromme T Klingenspor M. Browning Attenuates Murine White Adipose Tissue Expansion During Postnatal Development. Biochim Biophys Acta (2013) 1831(5):960–8. doi: 10.1016/j.bbalip.2013.01.016 23376694

[14] Birnbacher L Maurer S Scheidt K Herzen J Pfeiffer F Fromme T. Electron Density of Adipose Tissues Determined by Phase-Contrast Computed Tomography Provides a Measure for Mitochondrial Density and Fat Content. Front Physiol (2018) 9(707):1–8. doi: 10.3389/fphys.2018.00707 29962958 PMC6013718

[15] Chabowska-Kita A Trabczynska A Korytko A Kaczmarek MM Kozak LP. Low Ambient Temperature During Early Postnatal Development Fails to Cause a Permanent Induction of Brown Adipocytes. FASEB J (2015) 29(8):3238–52. doi: 10.1096/fj.15-271395 PMC4511198 25896784

[16] Chi J Lin Z Barr W Crane A Ge ZX Cohen P. Early Postnatal Interactions Between Beige Adipocytes and Sympathetic Neurites Regulate Innervation of Subcutaneous Fat. Elife (2021) 10:1–21. doi: 10.7554/eLife.64693 PMC7990502 33591269

[17] Wu Y Kinnebrew MA Kutyavin VI Chawla A. Distinct Signaling and Transcriptional Pathways Regulate Peri-Weaning Development and Cold-Induced Recruitment of Beige Adipocytes. Proc Natl Acad Sci USA (2020) 117(12):6883–9. doi: 10.1073/pnas.1920419117 PMC7104269 32139607

[18] Wang Y Paulo E Wu D Wu Y Huang W Chawla A. Adipocyte Liver Kinase B1 Suppresses Beige Adipocyte Renaissance Through Class IIa Histone Deacetylase 4. Diabetes (2017) 66(12):2952–63. doi: 10.2337/db17-0296 PMC5697944 28882900

[19] Giralt M Martin I Iglesias R Vinas O Villarroya F Mampel T. Ontogeny and Perinatal Modulation of Gene Expression in Rat Brown Adipose Tissue: Unaltered Iodothyronine 5′-Deiodinase Activity Is Necessary for the Response to Environmental Temperature at Birth. Eur J Biochem (1990) 193(1):297–302. doi: 10.1111/j.1432-1033.1990.tb19336.x 2171932

[20] Negron SG Ercan-Sencicek AG Freed J Walters M Lin Z. Both Proliferation and Lipogenesis of Brown Adipocytes Contribute to Postnatal Brown Adipose Tissue Growth in Mice. Sci Rep (2020) 10(1):1–11. doi: 10.1038/s41598-020-77362-x 33230135 PMC7683731

[21] Wang QA Tao C Gupta RK Scherer PE. Tracking Adipogenesis During White Adipose Tissue Development, Expansion and Regeneration. Nat Med (2013) 19(10):1338–44. doi: 10.1038/nm.3324 PMC4075943 23995282

[22] Hudak CS Gulyaeva O Wang Y Park S Lee L Kang C. Pref-1 Marks Very Early Mesenchymal Precursors Required for Adipose Tissue Development and Expansion. Cell Rep (2014) 8(3):678–87. doi: 10.1016/j.celrep.2014.06.060 PMC4138044 25088414

[23] Han J Lee JE Jin J Lim JS Oh N Kim K. The Spatiotemporal Development of Adipose Tissue. Development (2011) 138(22):5027–37. doi: 10.1242/dev.067686 22028034

[24] Kozak LP Newman S Chao PM Mendoza T Koza RA. The Early Nutritional Environment of Mice Determines the Capacity for Adipose Tissue Expansion by Modulating Genes of Caveolae Structure. PloS One (2010) 5(6):e11015. doi: 10.1371/journal.pone.0011015 20574519 PMC2888576

[25] Birsoy K Berry R Wang T Ceyhan O Tavazoie S Friedman JM. Analysis of Gene Networks in White Adipose Tissue Development Reveals a Role for ETS2 in Adipogenesis. Development (2011) 138(21):4709–19. doi: 10.1242/dev.067710 PMC3190384 21989915

[26] Chen HC Farese RV. Determination of Adipocyte Size by Computer Image Analysis. J Lipid Res (2002) 43(6):986–9. doi: 10.1016/S0022-2275(20)30474-0 12032175

[27] van Beek L van Klinken JB Pronk ACM van Dam AD Dirven E Rensen PCN. The Limited Storage Capacity of Gonadal Adipose Tissue Directs the Development of Metabolic Disorders in Male C57Bl/6J Mice. Diabetologia (2015) 58(7):1601–9. doi: 10.1007/s00125-015-3594-8 PMC4473015 25962520

[28] Gawronska-Kozak B Staszkiewicz J Gimble JM Kirk-Ballard H. Recruitment of Fat Cell Precursors During Long-Term High Fat Diet in C57BL/6J Mice Is Fat Depot Specific. Obesity (2014) 22(4):1091–102. doi: 10.1002/oby.20671 PMC5886012 24795999

[29] Rosenwald M Perdikari A Rülicke T Wolfrum C. Bi-Directional Interconversion of Brite and White Adipocytes. Nat Cell Biol (2013) 15(6):659–67. doi: 10.1038/ncb2740 23624403

[30] Vitali A Murano I Zingaretti MC Frontini A Ricquier D Cinti S. The Adipose Organ of Obesity-Prone C57BL/6J Mice Is Composed of Mixed White and Brown Adipocytes. J Lipid Res (2012) 53(4):619–29. doi: 10.1194/jlr.M018846 PMC3307639 22271685

[31] Kodde A Engels E Oosting A van Limpt K van der Beek EM Keijer J. Maturation of White Adipose Tissue Function in C57BL/6j Mice From Weaning to Young Adulthood. Front Physiol (2019) 10(836):1–14. doi: 10.3389/fphys.2019.00836 31354508 PMC6629938

[32] Roth CL Molica F Kwak BR. Browning of White Adipose Tissue as a Therapeutic Tool in the Fight Against Atherosclerosis. Metabolites (2021) 11(319):1–20. doi: 10.3390/metabo11050319 PMC8156962 34069148

[33] Cui X Nguyen NLT Zarebidaki E Cao Q Li F Zha L. Thermoneutrality Decreases Thermogenic Program and Promotes Adiposity in High-Fat Diet-Fed Mice. Physiol Rep (2016) 4(10):1–14. doi: 10.14814/phy2.12799 PMC4886167 27230905

[34] Moser C Straub LG Rachamin Y Dapito DH Kulenkampff E Ding L. Quantification of Adipocyte Numbers Following Adipose Tissue Remodeling. Cell Rep (2021) 35(4):1–11. doi: 10.1016/j.celrep.2021.109023 33909996

[35] Rosen ED MacDougald OA. Adipocyte Differentiation From the Inside Out. Nat Rev Mol Cell Biol (2006) 7(12):885–96. doi: 10.1038/nrm2066 17139329

[36] Pittenger MF Mackay AM Beck SC Jaiswal RK Douglas R Mosca JD. Multilineage Potential of Adult Human Mesenchymal Stem Cells. Science (80) (1999) 284:143–7. doi: 10.1126/science.284.5411.143 10102814

[37] Rangwala SM Lazar MA. Transcriptional Control of Adipogenesis. Annu Rev Nutr (2000) 20:535–59. doi: 10.1146/annurev.nutr.20.1.535 10940345

[38] Arner E Westermark PO Spalding KL Britton T Rydén M Frisén J. Adipocyte Turnover: Relevance to Human Adipose Tissue Morphology. Diabetes (2010) 59(1):105–9. doi: 10.2337/db09-0942 PMC2797910 19846802

[39] White U Ravussin E. Dynamics of Adipose Tissue Turnover in Human Metabolic Health and Disease HHS Public Access. Diabetologia (2019) 62(1):17–23. doi: 10.1007/s00125-018-4732-x 30267179 PMC6476187

[40] Guerra C Koza RA Yamashita H Walsh K Kozak LP. Emergence of Brown Adipocytes in White Fat in Mice Is Under Genetic Control Effects on Body Weight and Adiposity. J Clin Invest (1998) 102(2):412–20. doi: 10.1172/JCI3155 PMC508900 9664083

[41] Himms-Hagen J Melnyk A Zingaretti MC Ceresi E Barbatelli G Cinti S. Multilocular Fat Cells in WAT of CL-316243-Treated Rats Derive Directly From White Adipocytes. Am J Physiol Cell Physiol (2000) 279:670–81. doi: 10.1152/ajpcell.2000.279.3.C670 10942717

[42] Ailhaud G Hauner H. Development of White Adipose Tissue. In: Dekker M, editor. Handbook of Obesity: Etiology and Pathophysiology, 2nd ed. New York: ISBN 978-3-319-52029-2 (2003). p. 481–514.

[43] Skála J Barnard T Lindberg O. Changes in Interscapular Brown Adipose Tissue of the Rat During Perinatal and Early Postnatal Development and After Cold Acclimation - II. Mitochondrial Changes. Comp Biochem Physiol (1970) 33:509–28. doi: 10.1016/0010-406x(70)90368-3 4315637

[44] Schlein C Fischer AW Sass F Worthmann A Tödter K Jaeckstein MY. Endogenous Fatty Acid Synthesis Drives Brown Adipose Tissue Involution. Cell Rep (2021) 34(2):1–12. doi: 10.1016/j.celrep.2020.108624 PMC8240962 33440156

[45] Mottillo EP Balasubramanian P Lee YH Weng C Kershaw EE Granneman JG. Coupling of Lipolysis and *De Novo* Lipogenesis in Brown, Beige, and White Adipose Tissues During Chronic β3-Adrenergic Receptor Activation. J Lipid Res (2014) 55(11):2276–86. doi: 10.1194/jlr.M050005 PMC4617130 25193997

[46] Almind K Manieri M Sivitz WI Cinti S Kahn CR. Ectopic Brown Adipose Tissue in Muscle Provides a Mechanism for Differences in Risk of Metabolic Syndrome in Mice. Proc Natl Acad Sci USA (2007) 104(7):2366–71. doi: 10.1073/pnas.0610416104 PMC1892979 17283342

[47] Collins S Daniel KW Petro AE Surwit RS. Strain-Specific Response to β3-Adrenergic Receptor Agonist Treatment of Diet-Induced Obesity in Mice. Endocrinology (1997) 138(1):405–13. doi: 10.1210/endo.138.1.4829 8977430

[48] Li Y Bolze F Fromme T Klingenspor M. Intrinsic Differences in BRITE Adipogenesis of Primary Adipocytes From Two Different Mouse Strains. Biochim Biophys Acta Mol Cell Biol Lipids (2014) 1841(9):1345–52. doi: 10.1016/j.bbalip.2014.06.003 24953778

[49] Kozak LP Koza RA Anunciado-Koza R Mendoza T Newman S. Inherent Plasticity of Brown Adipogenesis in White Fat of Mice Allows for Recovery From Effects of Post-Natal Malnutrition. PloS One (2012) 7(2):1–13. doi: 10.1371/journal.pone.0030392 PMC3286483 22383960

[50] Liang X Yang Q Zhang L Maricelli JW Rodgers BD Zhu MJ. Maternal High-Fat Diet During Lactation Impairs Thermogenic Function of Brown Adipose Tissue in Offspring Mice. Sci Rep (2016) 6(34345):1–12. doi: 10.1038/srep34345 27686741 PMC5043374

[51] De Almeida DL Fabrício GS Trombini AB Pavanello A Tófolo LP Da Silva Ribeiro TA. Early Overfeed-Induced Obesity Leads to Brown Adipose Tissue Hypoactivity in Rats. Cell Physiol Biochem (2013) 32(6):1621–30. doi: 10.1159/000356598 24335411

[52] Xiao QX Williams SM Grayson BE Glavas MM Cowley MA Smith MS. Excess Weight Gain During the Early Postnatal Period Is Associated With Permanent Reprogramming of Brown Adipose Tissue Adaptive Thermogenesis. Endocrinology (2007) 148(9):4150–9. doi: 10.1210/en.2007-0373 17525123

[53] Fan R Toney AM Jang Y. Maternal N-3 PUFA Supplementation Promotes Fetal Brown Adipose Tissue Development Through Epigenetic Modifications in C57BL/6 Mice. Biochim Biophys Acta Mol Cell Biol Lipids (2018) 1863(12):1488–97. doi: 10.1016/j.bbalip.2018.09.008 PMC6203645 30266429

[54] Cao Q Jing J Cui X Shi H Xue B. Sympathetic Nerve Innervation Is Required for Beigeing in White Fat. Physiol Rep (2019) 7(6):1–7. doi: 10.14814/phy2.14031 PMC6418318 30873754

[55] Wu R Yu W Fu L Li F Jing J Cui X. Postnatal Leptin Surge Is Critical for the Transient Induction of the Developmental Beige Adipocytes in Mice. Am J Physiol Endocrinol Metab (2020) 318(4):E453–61. doi: 10.1152/ajpendo.00292.2019 PMC7191411 31961706

[56] Chabowska-Kita A Kozak LP. The Critical Period for Brown Adipocyte Development: Genetic and Environmental Influences. Obesity (2016) 24(2):283–90. doi: 10.1002/oby.21376 PMC4744992 26813522

